# Expression and secretion of glycosylated barley oxalate oxidase in *Pichia pastoris*

**DOI:** 10.1371/journal.pone.0285556

**Published:** 2023-05-11

**Authors:** William Donelan, ShiWu Li, Paul R. Dominguez-Gutierrez, Augustus Anderson IV, Li-Jun Yang, Cuong Nguyen, Benjamin K. Canales

**Affiliations:** 1 Department of Urology, College of Medicine, University of Florida, Gainesville, Florida, United States of America; 2 Department of Pathology, Immunology and Laboratory Medicine, College of Medicine, University of Florida, Gainesville, Florida, United States of America; 3 Department of Infectious Diseases and Immunology, College of Veterinary Medicine, University of Florida, Gainesville, Florida, United States of America; National Research Centre, EGYPT

## Abstract

Oxalate oxidase is an enzyme that degrades oxalate and is used in commercial urinary assays to measure oxalate levels. The objective of this study was to establish an enhanced expression system for secretion and purification of oxalate oxidase using *Pichia pastoris*. A codon optimized synthetic oxalate oxidase gene derived from *Hordeum vulgare* (barley) was generated and cloned into the pPICZα expression vector downstream of the N-terminal alpha factor secretion signal peptide sequence and used for expression in *P*. *pastoris* X-33 strain. A novel chimeric signal peptide consisting of the pre-OST1 sequence fused to pro-αpp8 containing several amino acid substitutions was also generated to enhance secretion. Active enzyme was purified to greater than 90% purity using Q-Sepharose anion exchange chromatography. The purified oxalate oxidase enzyme had an estimated Km value of 256μM, and activity was determined to be 10U/mg. We have developed an enhanced oxalate oxidase expression system and method for purification.

## Introduction

Oxalate oxidase (OxOx) catalyzes the oxidation of oxalate, reducing dioxygen to hydrogen peroxide and carbon dioxide (oxalate + O_2_ + 2 H^+^ ⇌ {\displaystyle \rightleftharpoons} → 2 CO_2_ + H_2_O_2_). It shows remarkable thermostability and resistance to proteases and have been identified in fungi, bacteria, and various plants with a great variance in biochemical structures and properties [1–14]. The plant enzymes have been most extensively studied and perhaps best characterized in barley and wheat. The enzyme is used clinically to determine oxalate levels from biological samples, primarily urine from kidney stone formers. The most common and commercially available OxOx enzyme available for purchase is derived from barley (Oxalate Reagent B, 591–2, Trinity Biotech, USA).

OxOx is detected in barley seedling roots during germination [15] and in the leaves of mature plants in response to infection [16]. Maximum enzymatic activity of purified barley OxOx has been shown at 35°C [17] and the reported pH optima ranges between pH 3.2–4.0 [17–20]. Regarding the thermostability, reports suggest that 80–100% of the activity remains when heated to 75°C and that the enzyme is extremely stable below 70°C [17, 21]. No loss of activity was observed when the enzyme was incubated for 4 hours in the presence of a large excess of trypsin [21]. There is agreement that the monomer ranges from 23-26kDa depending on the nature of glycans added during post-translational modification; however, the structure of the active oligomer has been reported as a dimer [17] and tetramer [21], was also assumed to be a pentamer based on characterization of the wheat homologue [22, 23], and most recently it has been identified as a hexamer based on X-ray crystallography studies [24]. EPR spectroscopy identified that the active form is a manganese-containing enzyme [19, 25], and the formation of disulfide bonds are also essential for enzymatic activity [13]. No studies have specifically characterized glycans for the barley OxOx enzyme, but studies for the highly similar homologue in wheat indicate the presence of N-linked but not O-linked glycans [22].

Active OxOx enzyme derived from *Hordeum vulgare* (barley) [25], *Triticum* (wheat) [26], and Ceriporiopsis subvermispora [27, 28] have been successfully expressed using *P*. *pastoris* expression systems. In a previous study, screening a cDNA expression library with polyclonal antibodies generated against purified barley OxOx from 10-day-old seedling roots and direct N-terminal amino acid sequencing was used to determine the mature 201 amino acid protein sequence of an active barley OxOx enzyme [29]. In this study, we have used a *P*. *pastoris* expression system to express this barley oxalate oxidase, and we developed methods to enhance enzyme secretion and purification.

## Materials and methods

### Plasmid construction

A DNA fragment was synthesized using gBlocks double stranded DNA synthesis (Integrated DNA Technologies) based on the amino acid sequence for a barley oxalate oxidase gene (NCBI Database, Accession: AAA32959.1). A codon optimized nucleic acid sequence with *P*. *pastoris* codon bias was generated that included 5’ and 3’ adapters to allow for ligation into the multiple cloning site of PICZαA (Invitrogen, Life Technologies) using the EcoRI and XbaI restriction sites. Plasmids were isolated using a plasmid purification kit (Qiagen).

### Protein expression and purification

We followed our previously published yeast protein expression protocols to express OxOx enzyme [30, 31]. In brief, the OxOx transformed *P*. *pastoris* competent cells were selected on YPD plates containing 100 μg/ml zeocin. After incubation for 2 to 3 days at 30°C, single zeocin-resistant colonies were selected for protein expression. Selected colonies were cultured in 5 ml YPD medium containing 100 μg/ml zeocin overnight under shaking (200 rpm 30°C). Then, 5 mL of yeast culture was transferred into flasks containing 50 mL YPD (1% yeast extract, 2% peptone, 2% dextrose, and 2% agar) medium containing 100 μg/ml zeocin and cultured under shaking for another 12 to 14 h. Scale-up expression was performed by transferring 50 ml yeast solution into 500 ml YPD medium containing 100 μg/ml zeocin and culturing overnight. After the A280 value reached 12 to 18, the cells were harvested by centrifugation (8000 rpm for 10 min) and resuspended in 100 ml buffered methanol-complex medium ((BMMY), 1% yeast extract, 2% peptone, 100 mM potassium phosphate (pH 6.0), 1.34% yeast nitrogen broth, 0.4 mg/L biotin and 0.5% methanol). Subsequently, the cells were incubated at 30°C for 4 days under shaking (200 rpm), and 0.5% methanol was added to the medium every day.

For purification, a strong anion exchange resin Q-Sepharose fast flow (GE Healthcare) was added to a column and equilibrated with 20mM Tris-HCl. 1M Tris-HCl buffer, pH 9.0 was added to dialyzed culture media for a final concentration of 20mM with approximate final pH 8.9. The column was washed with 20mM Tris-HCl. Isocratic elution was performed using 20mM Tris-HCl wash buffer containing 1M NaCl and eluent was dialyzed overnight against diH_2_O with <6-8kDa dialysis tubing.

### Western blotting

Samples were loaded into precast 4–20%SDS-PAGE gels (BioRad) and transferred onto PVDF membranes. The membranes were blocked with 5% skim milk in Tris-buffered saline, pH 7.4 containing 0.05% Tween 20 (PBST), at room temperature for 1 h, and were incubated with primary anti-barley oxalate oxidase 1 antibody (Origene, AP21356AF-N, rabbit polyclonal, 1:200 dilution) at 4°C overnight. Membranes were washed with PBST and a secondary anti-rabbit IgG antibody conjugated to horseradish peroxidase (HRP; Cell Signaling Technology, #7074, 1:1000 dilution) was incubated at room temperature for 1 h, and then washed with PBST. HRP signal was activated using SuperSignal West Pico PLUS Chemiluminescent Substrate (ThermoFisher Scientific) and Images were taken using an ImageQuant LAS 4000 imager (GE Healthcare). The antibodies were diluted to their appropriate ratio according to manual instructions. Experiments were performed in triplicate and a representative blot was shown. Original uncropped and unadjusted blot and gel images are shown in S1 File.

### Enzyme kinetic assays

Enzyme activity and kinetics were performed using the Trinity Biotech Oxalate Assay Kit with modifications. Reagent B, containing the Trinity OxOx and HRP, was not used. Instead, HRP purchased from Millipore-Sigma was used with our expressed OxOx enzyme. In brief, oxalate is oxidized with our enzyme to generate carbon dioxide and hydrogen peroxide. The hydrogen peroxide then reacts with 3-methyl-2-benzothiazolinone hydrazone and 3-(dimethylamino) benzoic acid in the presence of HRP to produce an indamine dye with an absorbance maximum at 590 nm. The color intensity is directly proportional to the concentration of oxalate in the sample. The oxalate concentrations used were in the micromolar range where the enzyme exhibits its highest activity and span slightly beyond the concentrations where the substrate (product) inhibition occurs. Data from samples experiencing significant substrate (product) inhibition were not used in the calculation of K_m_. The data set is available in S2 File.

### Enzyme activity assay

The enzyme activity assay was performed similarly to the kinetic assays with the addition of a H2O2 standard curve in the range of 100 pmol– 5nmol per well. Absorbance (Abs) at 590nm was measured every 30sec per well at 37°C for optimal enzyme activity, and the reader plate was shaken between read steps for more accurate results. A reaction without oxalate was used to subtract background oxidation. The data set is available in S2 File.

## Results

**Fig 1:**
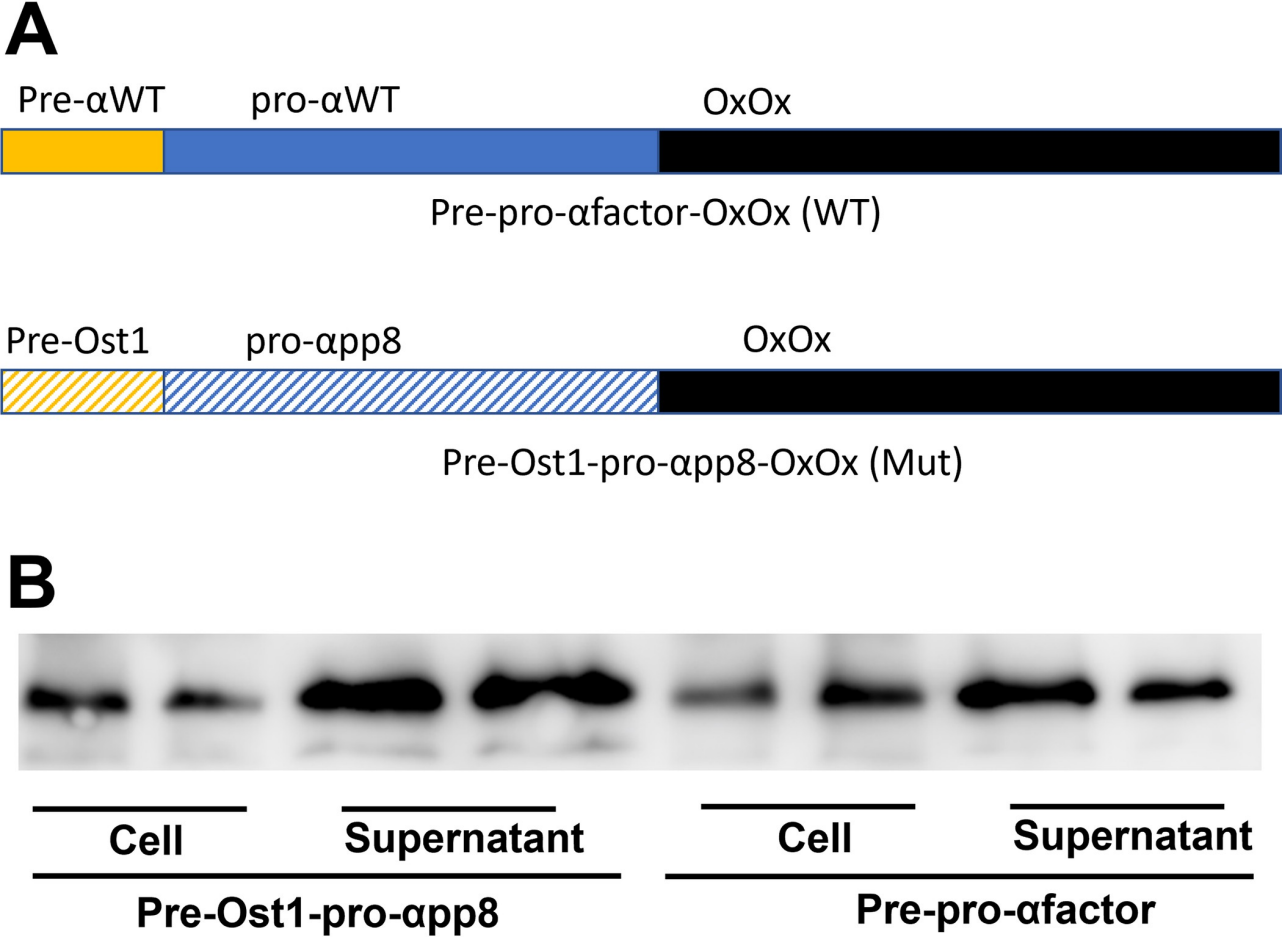
Effects of OST1-αpp8 fusion leader sequence on OxOx secretion in *P*. *pastoris*. (A) Depiction of wild type pre-pro αfactor-OxOx gene and Pre-Ost1-pro-αpp8-OxOx genes. (B) Western blot using supernatants from individual yeast clones following transformation with OxOx expression plasmids with indicated wild type or mutant leader sequences.

Based on the amino acid sequence for the barley oxalate oxidase gene (NCBI Database, Protein: GI 289357) [29], we generated a codon optimized synthetic DNA fragment which was cloned into the pPICZalpha expression vector containing the N terminal alpha factor sequence. We also generated a construct in which we replaced the wild type alpha factor N-ter sequence with a novel leader sequence (Fig 1A). This chimeric signal peptide consisted of the pre-OST1 sequence [32] fused to pro-αpp8 containing several amino acid substitutions (S1 Fig) that were previously determined to improve secretion [33]. The *P*. *pastoris* X-33 yeast strain was transformed, and individual clones were cultured and induced for enzyme expression. As demonstrated by western blotting, this novel chimeric leader sequence reduced the cellular level and enhanced secretion of OxOx enzyme into the culture media compared to the wild type alpha factor sequence (Fig 1B).

**Fig 2:**
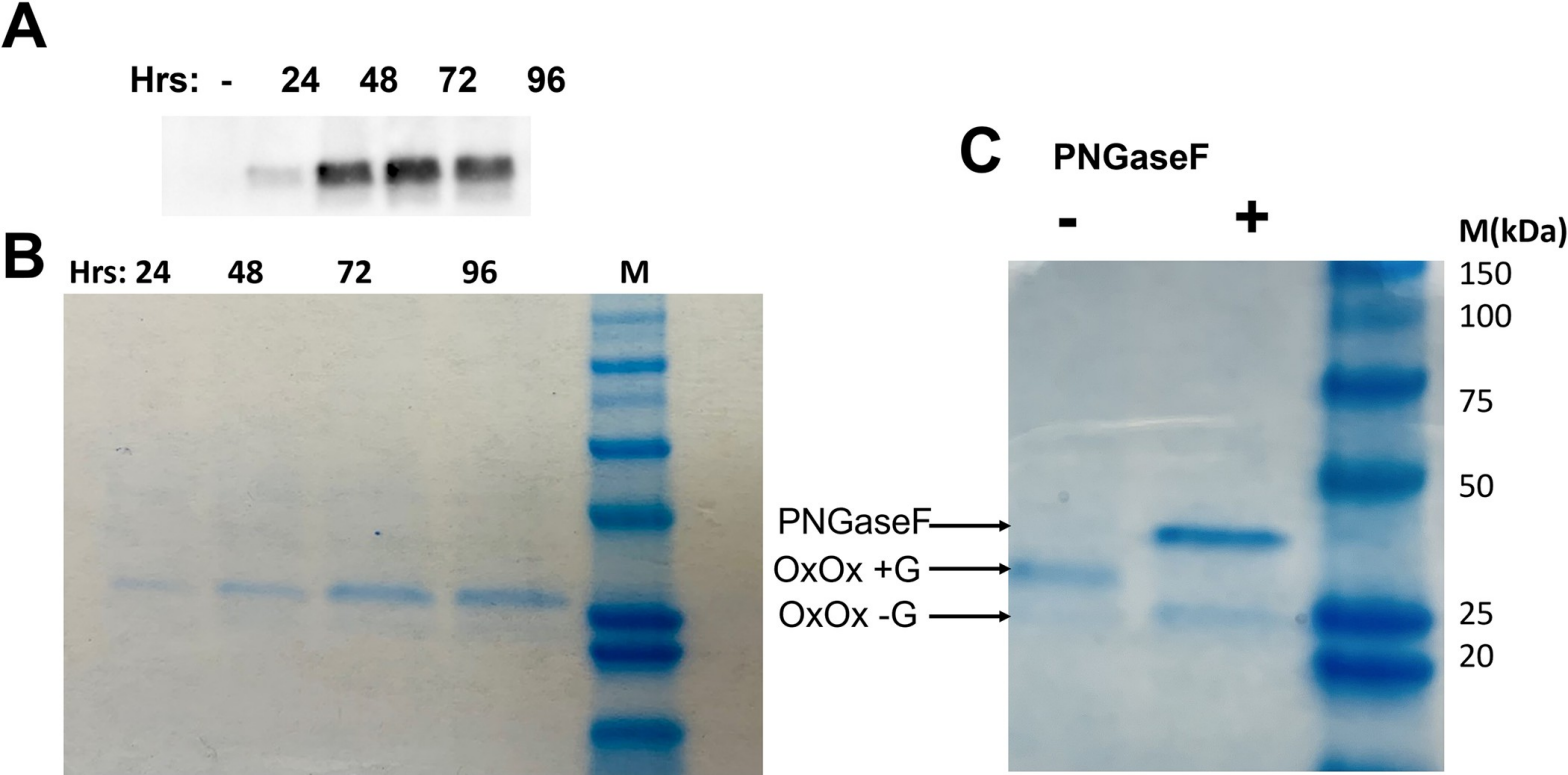
Expression characteristics of OxOx in *P*. *pastoris*. (A) Supernatants from representative yeast clones were collected at indicated time points and subjected to western blotting. (B) Supernatants from representative yeast clones were collected at indicated time points and subjected to Coomassie staining for total protein following acrylamide gel electrophoresis. (C) Supernatant from representative yeast clones was collected at 96 hours, treated with or without PNGase F, and stained with Coomassie following acrylamide gel electrophoresis (G = glycan).

We next conducted an expression time-course analysis. Enzyme expression was induced for 4 days, and samples of the culture media were taken daily and probed with an anti-OxOx antibody. We saw a high, sustained level of expression by three days post-induction for transformants that successfully expressed enzyme (Fig 2A). Pre-purification Coomassie staining for total protein, showed a band with molecular weight greater than 25kDa indicating a heavily glycosylated OxOx protein (Fig 2B). The molecular weight was reduced by removing the glycan following digestion with PNGaseF (Fig 2C).

**Fig 3:**
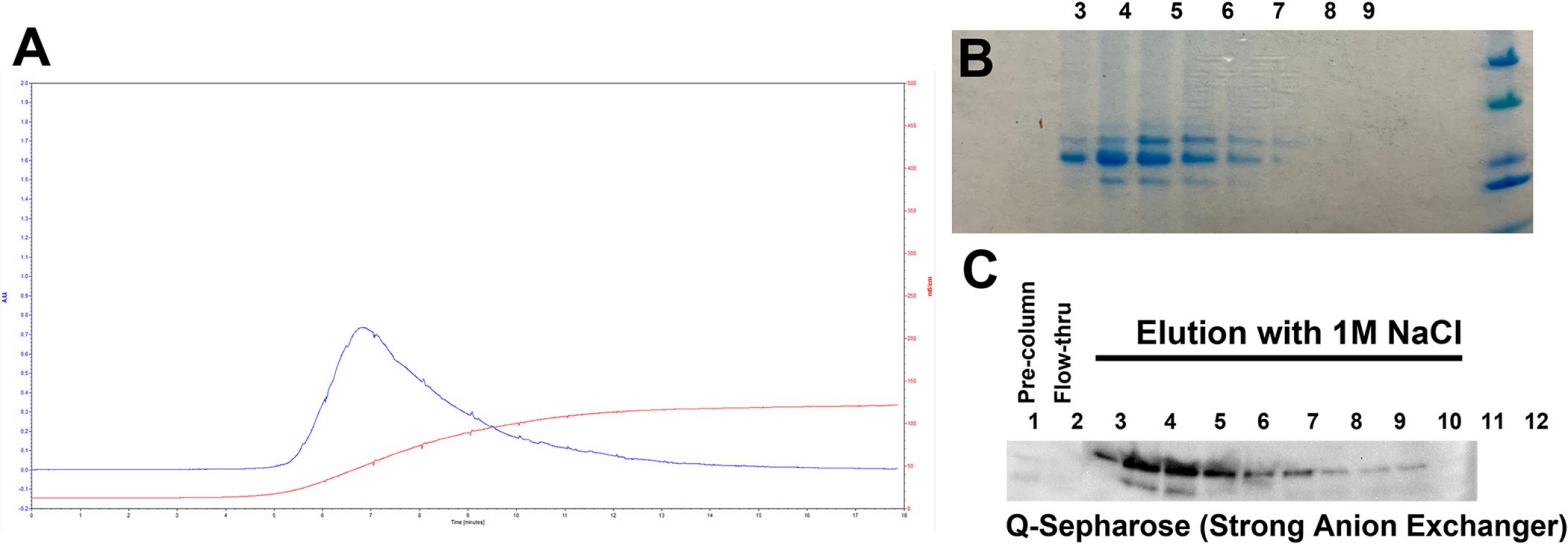
Purification of OxOx using anion exchange chromatography. (A) Chromatographic profile of OxOx purification. The blue line represents protein A280 and the red line represents electrical conductivity (mS/cm). 1 ml elution fractions were subjected to (B) Coomassie staining for total protein or (C) western blotting following acrylamide gel electrophoresis.

Using the SIB Swiss Institute of Bioinformatics tool Expasy, we determined the isoelectric point of the OxOx enzyme to be approximately 5.5, and thus used anion exchange chromatography for purification. An alkaline Tris buffer was used to induce a strong negative charge for binding to Q-Sepharose resin, and enzyme eluted well with a 1M NaCl buffer (Fig 3A). Post-purification Coomassie staining for total protein, showed a highly enriched OxOx band (Fig 3B) that was also seen by western blotting (Fig 3C).

**Fig 4:**
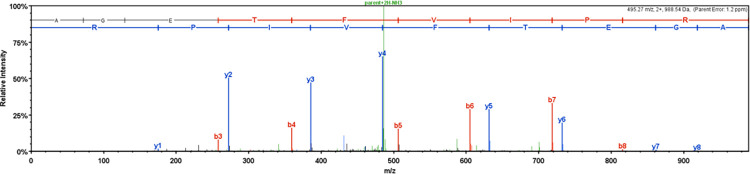
LC–MS/MS spectrum. A single OxOx band was excised from a Coomassie gel and protein was digested with trypsin. The peptide “AGETFVIPR” was identified with 100% accuracy that is unique to the barley-derived oxalate oxidase protein and that is not present in the *P*. *pastoris* proteome.

No enzyme was visibly observable in the flow through by western blot analysis. Following trypsin digestion, liquid chromatography tandem mass spec analysis was used to identify a peptide (AGETFVIPR) with 100% accuracy that is unique to our enzyme and not present in the yeast proteome (Fig 4).

**Fig 5:**
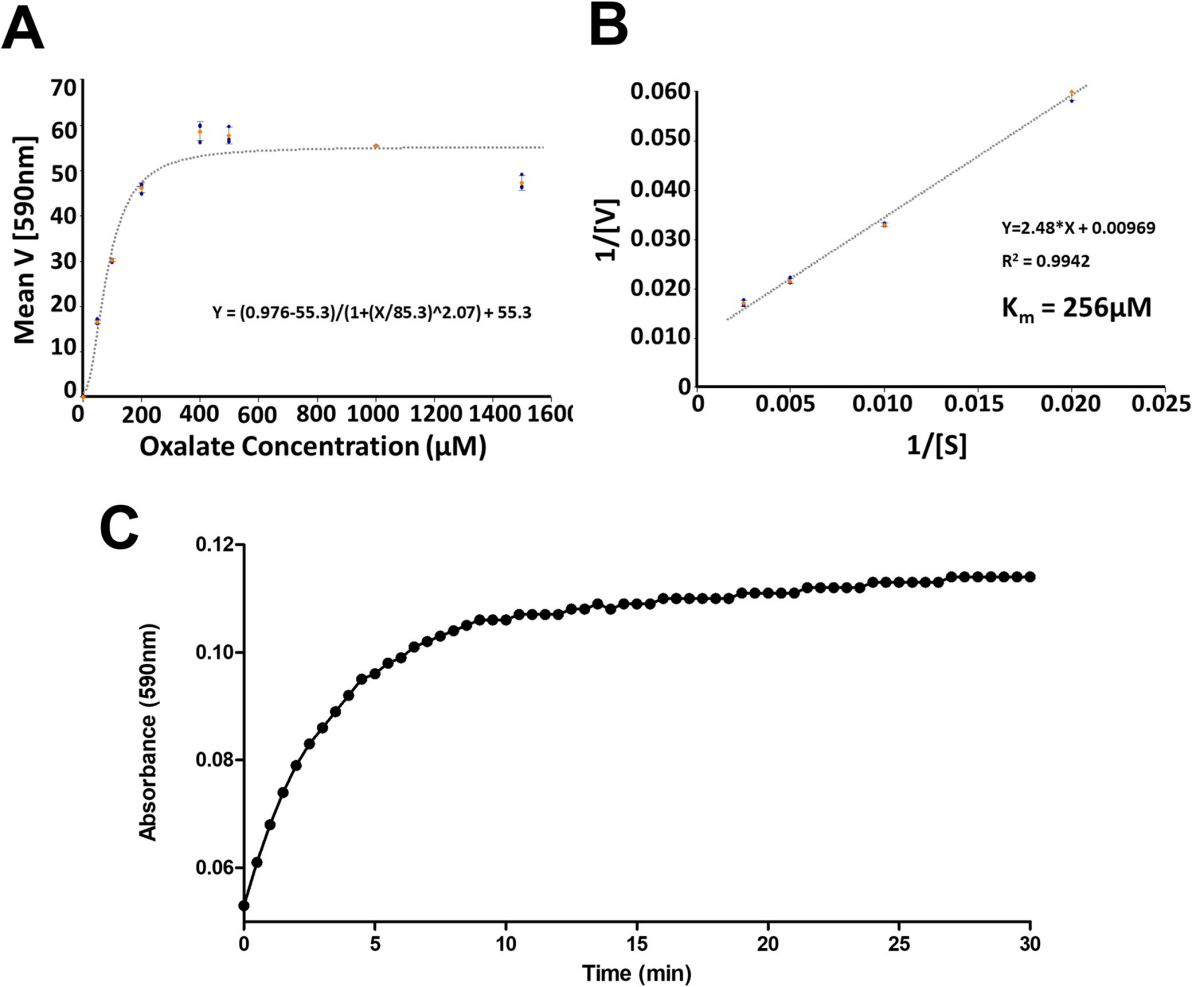
Reaction kinetics and activity of OxOx. (A) Michaelis-Menten Saturation Curve. Kinetic analysis was performed using a plate reader at 37°C. Purified OxOx (400μg/ml) was added to buffer pH 3.1 containing (MBTH, 0.11mM, DMAB, 1.6mM, HRP, 0.1U/ml) at designated potassium oxalate concentrations. Absorbance at 590nm was measured every 30 sec for 30 min to determine the rate of reaction which was plotted against substrate concentration. (B) Lineweaver-Burk Plot. Since the purified OxOx displayed Michaelis-Menten kinetics for concentrations up to 400μM, we plotted a linear transformation of the rate against substrate concentrations and estimate the enzyme Km = 256μM. (C) Purified OxOx activity was determined using conditions similar to kinetic assays and determined using a H_2_O_2_ standard curve.

The reaction rate at various substrate concentrations was determined to generate a Michaelis-Menten saturation curve (Fig 5A), and a Lineweaver-Burke plot was used to calculate a Km of 256uM (Fig 5B), which is within the range of similar enzymes reported in the literature [18, 34, 35]. Kinetic analysis confirmed successful expression of a bioactive OxOX enzyme. OxOx protein concentration was determined using the bicinchoninic acid (BCA) assay and showed a yield of 3.3mg/L. Enzyme activity was determined to be 10U/mg by OxOx assay using a H_2_O_2_ standard curve (Fig 5C).

**Fig 6:**
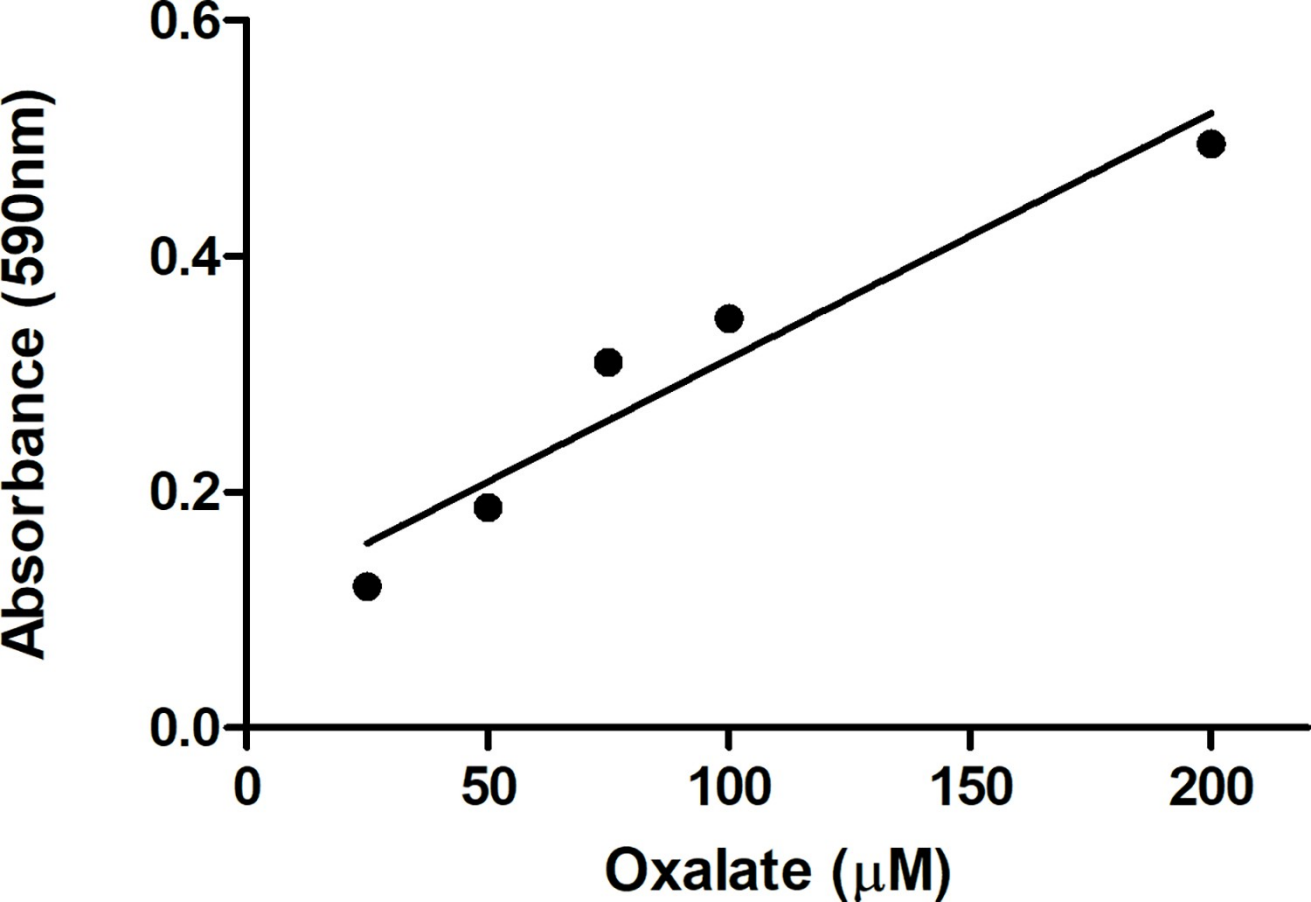
Determination of oxalate concentration in human urine. The oxalate concentration of a human urine sample was first measured using the Trinity Biotech Oxalate Assay. Several dilutions of this human urine were then combined with purified OxOx and buffer pH 3.1 containing (MBTH, 0.11mM, DMAB, 1.6mM, HRP, 0.1U/ml). Reactions were incubated for 10 min at 37°C and absorbance was measured at 590nm.

Finally, we used the yeast OxOx enzyme to determine the concentration of oxalate in a human urine sample (Fig 6). The concentration of oxalate in the urine was previously determined using the Trinity Biotech Oxalate Assay. We made several dilutions of the urine and measured the oxalate concentration using similar assay conditions as for the kinetic assays. We were able to correctly identify the oxalate concentration in the undiluted urine, and reasonable linearity was found when measuring diluted samples (R2 = 0.9286). Thus, our enzyme is suitable for measuring the concentration of oxalate from human urine.

## Discussion

The *P*. *pastoris* yeast expression system is one of the most widely used tools for the expression of recombinant proteins [36]. Although many proteins have been successfully expressed and secreted using this system, high level secretion remains a common problem in some cases [37]. Secretory leader sequences are made of two distinct regions, pre and pro. The process of protein translocation is grouped into two categories and is mainly dependent on the pre region of the leader sequence. Cotranslational is the classification in which this process occurs during translation, whereas posttranslational occurs after translation is complete and the protein is released from the ribosome. Most secreted and membrane-bound proteins are cotranslationally translocated [38]. The *Saccharomyces cerevisiae* alpha factor secretion signal peptide widely used by the *P*. *pastoris* expression system uses the posttranslational process [39]. As a result, proteins that can fold in the cytosol may be poorly translocated across the ER membrane and fail to enter the secretory pathway. Another problem that can lead to reduced secretion of recombinant proteins, especially so for self-associating proteins, is protein aggregation caused by the pro region of the signal peptide. Barrow et. al found that replacing the pre region of the alpha factor with the signal sequence OST1 substantially improved for certain proteins [32]. They also found that an allelic variant of the alpha factor pro region protein aggregation in the ER. In a study by Rakestraw et. al, a directed evolutionary approach was taken to identify leader sequences that enhance secretion of heterologous protein [33]. In our study we utilized a chimeric leader sequence combining elements from these previous studies to enhance the expression of OxOx in our system.

OxOx plays an important role in healthcare for oxalate-related diseases. Roughly 80% of kidney stones are calcium based, and the majority of these contains a mixture of both calcium and oxalate [40]. Kidney stones are a major health care problem in the United States and the world. Kidney stone disease is a common, costly, and painful disorder that can lead to renal failure and death. The prevalence of symptomatic nephrolithiasis in the US is 8.8%, exceeding other common diseases such as diabetes (8.2%), chronic obstructive pulmonary disease (6.3%), and stroke (3%) [41, 42]. While oxalate is produced endogenously within the liver from ascorbic acid and collagen metabolism, up to 90% of urinary oxalate originates through gut absorption from the diet, especially from oxalate-containing plant-based foods (e.g., spinach, bran, leafy greens) or plant products (e.g., chocolate, almonds, peanut butter) [43].

Assessing oxalate in urine is a critical step for treating and monitoring kidney stones [44]. To estimate stone risk and assist with stone prevention, urologist frequently ask stone formers to collect a 24-hour urine to measure mineral and electrolyte constituents, including oxalate. Current methods for determining oxalate are relatively complex, labor intensive, and require specialized equipment in laboratory settings. Development of an oxalate dipstick test (similar to a urine glucose dipstick) that can quickly and accurately quantify the amount of oxalate in urines and be used to self-monitor could possibly improve the current standard of care. Unfortunately, there is no simple and quick point-of-care oxalate test for clinicians and patients. One of the major hurdles is the lack of commercially viable source of OxOx needed to manufacture such a test. Industrial production of OxOx using a large-scale yeast expression system could provide a viable means of OxOx enzyme for such a test. Our enhanced methods for expression and purification of OxOx may be of interest for such applications.

## Conclusions

We have established an enhanced expression system for OxOx using a *Pichia pastoris* expression system and developed methods for enzyme purification. OxOx enzyme was expressed at 3.3mg/L with an activity of 10U/mg. In future studies we may focus on upscaling production from our current batch culture conditions to further increase the yield and activity of OxOx enzyme produced.

## Supporting information

S1 Fig Amino acid substitutions for pro-αpp8. (TIF)

S1 File PDF file containing original uncropped and unadjusted blot and gel images. (PDF)

S2 File Excel file containing the dataset. (XLSX)
