# Supplementary figures and images for: Expression and secretion of glycosylated barley oxalate oxidase in Pichia pastoris
Source: PLoS One. 2023 May 11;18(5):e0285556. doi: 10.1371/journal.pone.0285556 (PMC10174515; doi:10.1371/journal.pone.0285556)

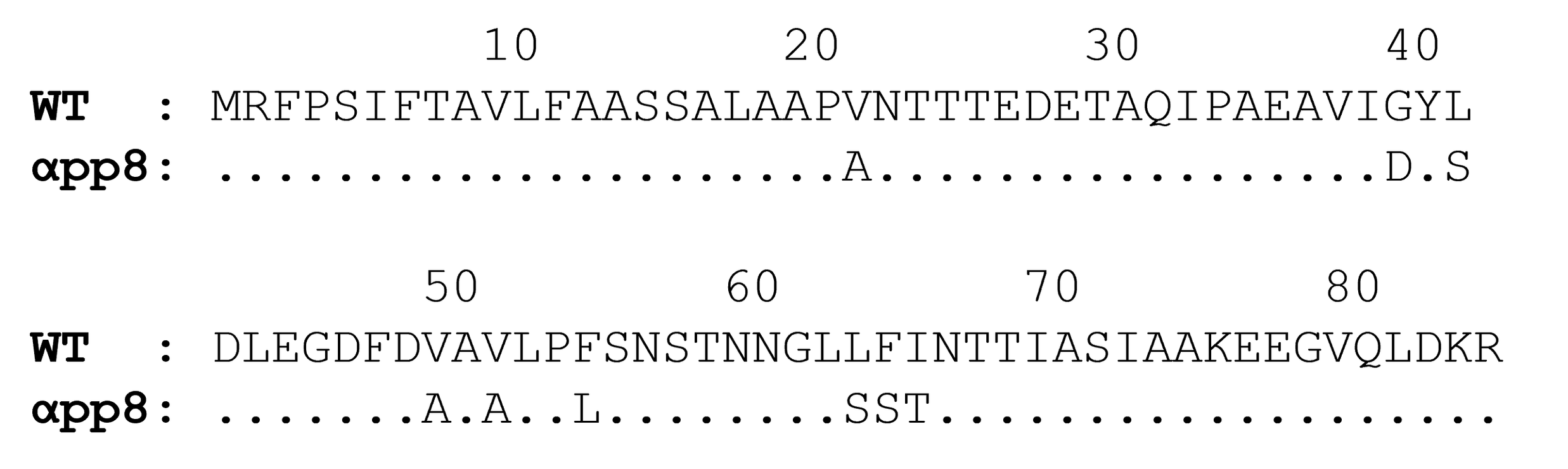

Supplement: S1 Fig — (TIF) [file pone.0285556.s001.tif]
