# Supplementary material for: Expression and secretion of glycosylated barley oxalate oxidase in Pichia pastoris
Source: PLoS One. 2023 May 11;18(5):e0285556. doi: 10.1371/journal.pone.0285556 (PMC10174515; doi:10.1371/journal.pone.0285556)
Supplement: S1 File — (PDF) [file pone.0285556.s002.pdf]

S1 File. PDF file containing original uncropped and unadjusted blot and gel images.

A

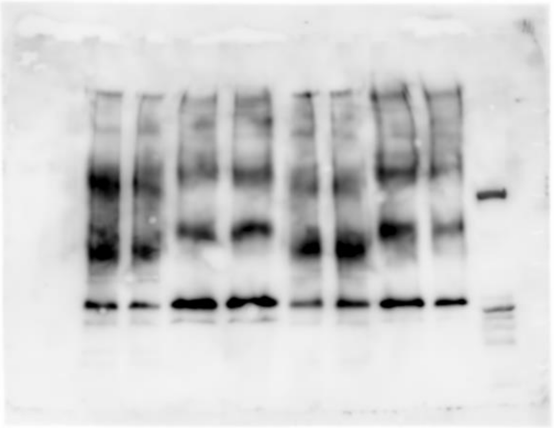

Fig 1B original western blot image taken using ImageQuant LAS 4000 imager (GE Healthcare). Lane 10 (Marker) not presented in manuscript.

B

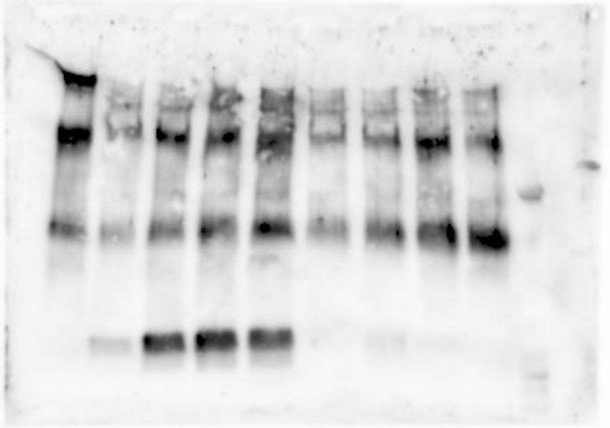

Fig 2A original western blot image taken using ImageQuant LAS 4000 imager (GE Healthcare). Lanes 6-10 not presented in manuscript.

C

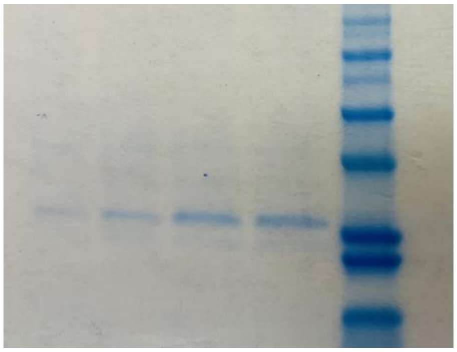

Fig 2B original Coomassie image taken using iPhone (Apple).

D

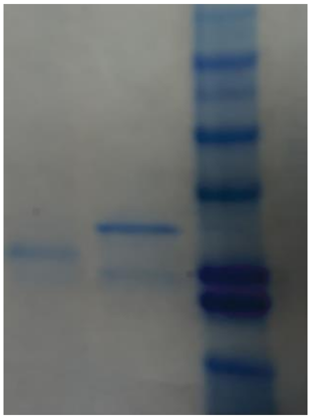

Fig 2C original Coomassie image taken using iPhone (Apple).

E

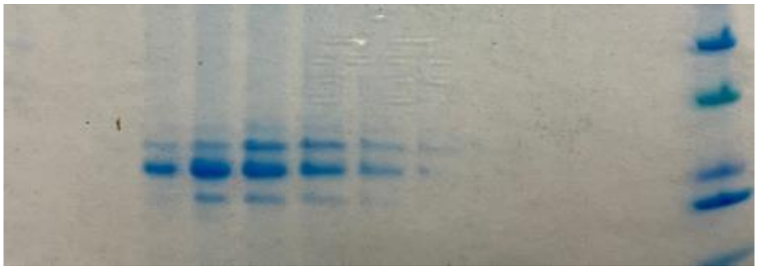

Fig 3B original Coomassie image taken using iPhone (Apple).

F

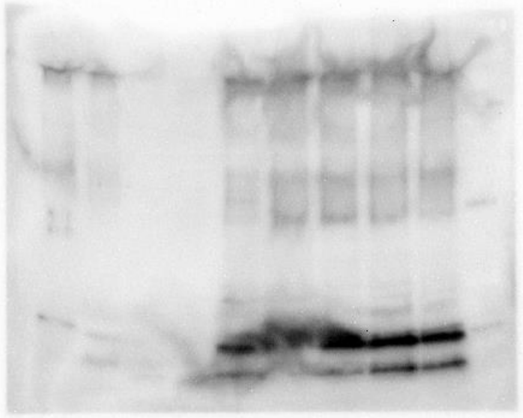

Fig 3C original western blot image taken using ImageQuant LAS 4000 imager (GE Healthcare). Lane 2 and 3 are shown in figure as pre-column and flow-thru, respectively. Lanes 1 and 4-10 not presented in manuscript.

G

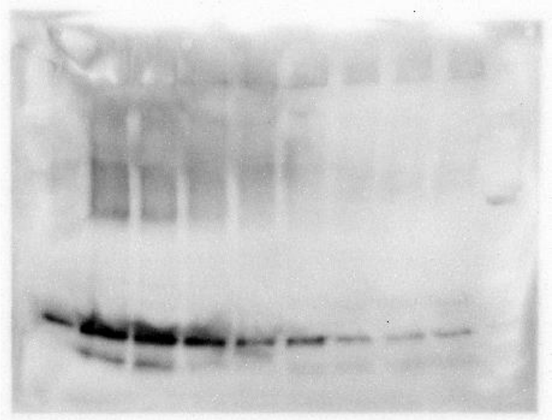

Fig 3C original western blot image taken using ImageQuant LAS 4000 imager (GE Healthcare). All 10 lanes in the blot are shown as lanes 3-12 in the figure.
